# Peripheral intravenous waveform analysis for evaluating volume status in healthy volunteers and mechanically ventilated patients

**DOI:** 10.1007/s10877-025-01408-6

**Published:** 2026-01-29

**Authors:** Aura Koistinaho, Sole Lindvåg Lie, Svein Aslak Landsverk, Harald Lenz, Marius Rehn, Jonny Hisdal, Lars Øivind Høiseth

**Affiliations:** 1 Institute of Clinical Medicine, University of Oslo, Oslo, Norway; 2 Department of Research and Development, Norwegian Air Ambulance Foundation, Oslo, Norway; 3 Akershus University Hospital, Akershus, Norway; 4 Division of Emergencies and Critical Care, Oslo University Hospital, Oslo, Norway; 5 Department of Vascular Surgery, Oslo University Hospital, Oslo, Norway

**Keywords:** Venous pressure, Hemodynamics, Lower body negative pressure, Hypovolemia

## Abstract

Timely diagnosis of blood loss and evaluation of intravascular volume status are pivotal tasks in clinical practice. Recent studies in animals and during lower body negative pressure (LBNP) in humans indicate that peripheral intravenous pressure waveform analysis (PIVA) may detect early stages of blood loss. As PIVA only requires a peripheral venous cannula, it may have value in emergency settings. However, its clinical relevance remains uncertain. This study examined how volume changes affect the PIVA-derived fundamental frequency (PIVA_F1_). Two cohorts were studied. The LBNP cohort comprised 15 healthy volunteers exposed to simulated blood loss in 10 mmHg increments of LBNP every two minutes from 0 to 80 mmHg, or until hemodynamic decompensation. The general anesthesia (GA)-cohort included 20 patients undergoing laparoscopic surgery who underwent preload increase with a head-down tilt. Peripheral intravenous pressure waveforms were continuously recorded from an antecubital vein and analyzed using short-time Fourier transform to extract the amplitude at the heart-rate frequency (PIVA_F1_). Changes in PIVA_F1_ were analyzed using linear regression. In the LBNP-cohort, data were log(e) – transformed and associated with a change per LBNP level of -0.11 (95% CI -0.14 to -0.09, *P* < 0.001). In the GA-cohort, PIVA_F1_ did not reliably predict a 10% change in stroke volume with head-down tilt [AUC 0.71 (95% CI 0.47 to 0.96; *P* = 0.11)]. We found statistically significant reductions in PIVA_F1_ during simulated blood loss but PIVA_F1_ did not predict increasing stroke volume during head-down tilt in general anesthesia. The clinical significance of PIVA remains to be elucidated.

## Introduction

Timely detection of blood loss and accurate evaluation of intravascular volume status remain challenging tasks, yet they are pivotal for optimizing patient management in surgical, acute, and intensive care settings. Standard hemodynamic variables such as heart rate and blood pressure are routinely used to monitor hemodynamic status and guide therapy in both emergency settings and during elective surgery. Due to compensatory mechanisms, these variables have a low diagnostic accuracy, making early recognition of hypovolemia difficult [1]. Advanced hemodynamic monitoring techniques are often invasive, operator-dependent or technically complex. Consequently, there is a high demand for less invasive, operator-independent point of care methods to detect volume depletion.

Peripheral venous pressure has been suggested to estimate volume status, substituting central venous pressure [2, 3]. Given the well-known limitations of central venous pressure in assessing volume status [4] peripheral venous pressure in itself is not likely to be of significant value. However, time-frequency peripheral intravenous pressure waveform analysis (PIVA) using pressure from a standard peripheral intravenous cannula has been explored as a method to assess changes in volume status [5, 6]. PIVA_F1_, estimating the amplitude of the fundamental frequency (F1) of the pulse-synchronous oscillations in peripheral venous pressure, has been associated with early blood loss in rodent [7] and porcine models [8]. Both these studies were performed in general anesthesia with controlled ventilation.

Lower body negative pressure is an experimental model for simulating blood loss in awake humans [9, 10]. Application of increasing levels of negative pressure causes blood pooling in the pelvis and lower extremities, leading to decreasing venous return and central hypovolemia. One study has explored the effects of LBNP on peripheral pressure waveform metrics [11]. Significant effects of LBNP on these metrics were found, but as the study used relatively large LBNP steps, a protocol with more gradual LBNP-application could provide further insight.

Head-down tilt involves a whole-body inclination that positions the head below the level of the lower limbs, resulting in a short-term increase in venous return. By evaluating the stroke volume response, it is widely used to assess volume status and fluid responsiveness [12, 13].

PIVA_F1_ has shown promising results for assessing volume status in animal models, but the method needs further validation in human studies if it is to be used clinically. Further, the effect of general anesthesia and controlled ventilation remains to be elucidated. We therefore performed the current analyses where we aimed to study PIVA_F1_ during (1) simulated blood loss in spontaneously breathing humans and (2) in mechanically ventilated patients in general anesthesia before performing a head-down tilt.

## Methods

Data from two previously published studies [14, 15] were analyzed. The studies were approved by the regional ethics committee (REC South East (www.rekportalen.no); references 2009/2181-1 and 95320, 1 April 2020) and the Data Protection Officer at Oslo University Hospital (date 12.05–2010 and reference no. 20/18284). One study was registered in clinicaltrials.gov (NCT04641949). All healthy volunteers and patients provided written informed consent.

### Study cohorts

For the present study, the two cohorts are termed the LBNP–cohort and the general anesthesia (GA)–cohort. The eligibility criteria for these two cohorts are presented in Table 1.

**Table 1:** Eligibility criteria

LBNP-cohort		GA-cohort
Inclusion criteria • Healthy volunteers aged 18–65 years Exclusion criteria • Any medical condition limiting physical exertional capacity or requiring regular medication use • Chronic pain • Pregnancy • Substance abuse • Use of analgesics or complementary medicine within the past 2 days or alcohol within 24 h prior to participating in the study		Inclusion criteria • Age > 18 years • Scheduled for laparoscopic gastrointestinal surgery in general anesthesia • Arterial and central venous catheterization and cardiac output-monitoring using esophageal doppler Exclusion criterion • Cardiac arrhythmias

LBNP, lower body negative pressure; GA, general anesthesia

The LBNP-cohort, comprising 15 healthy volunteers, was exposed to progressive LBNP. In the original study [15], three LBNP- runs were performed for each subject on separate visits to compare the effects of two different drugs to placebo. To exclude the effects of the drugs, only data from the placebo visits were used for the present analysis.

The GA-cohort comprised 20 patients scheduled to undergo laparoscopic gastrointestinal surgery in general anesthesia [14]. In the original study, dynamic variables of fluid responsiveness were explored with head-down tilts before and after establishment of pneumoperitoneum. For the present study, only data obtained before pneumoperitoneum were analyzed.

### LBNP protocol

**Fig. 1:**
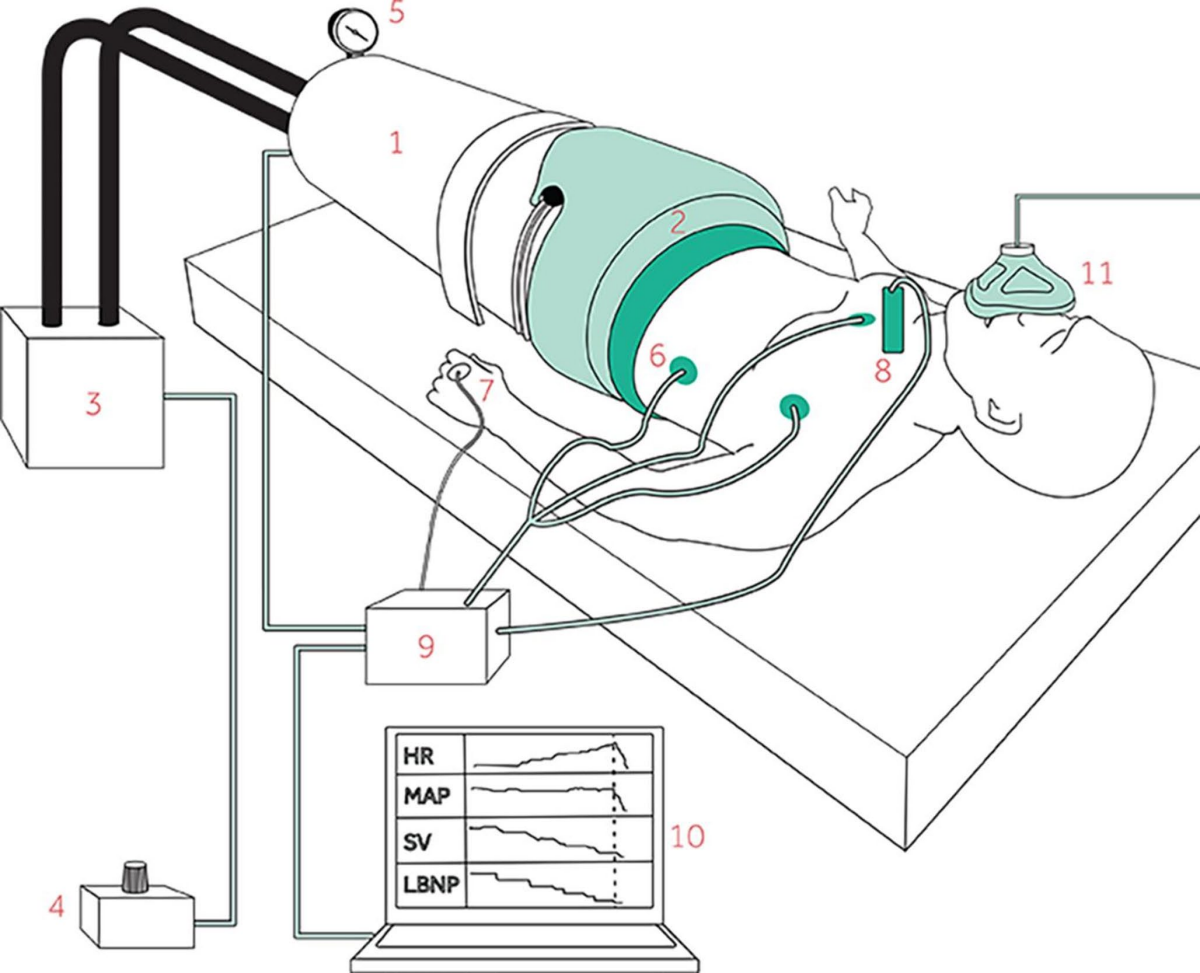
Illustration showing lower body negative pressure (LBNP), graphics reused from protocol under CC BY 4.0 license [16]. The participant was positioned inside (1) the LBNP chamber which was sealed (2) under the level of iliac crest and connected to a vacuum pump (3) controlled by (4) pressure control unit. The chamber was displayed on (5) a pressure monitor, Hemodynamic variables (6–8) including heart rate, mean arterial pressure, stroke volume and peripheral venous pressure were transmitted to (9) a data acquisition device and sampled on a computer continuously (10). Expired CO_2_ was measured by side stream capnography (11)

Participants were positioned in the LBNP chamber (illustrated in Fig. 1) sealed at the level of the iliac crest using a neoprene skirt. Measurements were recorded over a 2-minute period at each LBNP level, with chamber pressure progressively decreased in 10 mmHg increments starting from 0 mmHg. The LBNP exposure was discontinued when LBNP 80 was completed, or earlier if decompensation occurred as defined by any of the following: Symptoms of imminent cardiovascular collapse (light-headedness, nausea, or sweating), reduction of MAP or heart rate to < 75% of baseline for > 3 s, or upon participant request for reasons other than the above.

Continuous non-invasive arterial pressure (Nexfin; BMEYE, Amsterdam, the Netherlands), stroke volume by suprasternal Doppler (SD-50; Vingmed Ultrasound, Horten, Norway) and heart rate obtained with ECG (BioAmp/PowerLab; ADInstruments, Bella Vista, NSW, Australia) were sampled at 1000 Hz in LabChart 8.1.9 (ADInstruments). A 22G peripheral venous cannula was inserted in an antecubital vein, and peripheral venous pressure waveform was measured continuously from this cannula (Marquette Solar 8000i; GE Medical Systems, Milwaukee, WI, USA) and sampled in LabChart (ADInstruments).

### GA-protocol

Following overnight fasting, the patients were premedicated with oral diazepam 5–10 mg. General anesthesia was induced with sodium thiopental and fentanyl after which cisatracurium was administrated. The patients were orally intubated and mechanical ventilation initiated (Dräger Primus; Drägerwerk AG & Co. KGaA, Lübeck, Germany) in volume-controlled mode with tidal volume 8.6 (0.8) mL/kg predicted body weight and a positive end expiratory pressure of 5 cm H_2_O. Anesthesia was maintained with sevoflurane.

Before commencing surgery, baseline registrations were performed for one minute in the horizontal, supine position. Thereafter, a head-down tilt was performed by tilting the operating table. After 15 s stabilization, cardiac stroke volume was measured and averaged over 30 s in the head-down position.

Pressure from a peripheral venous cannula placed in an antecubital vein was sampled from the monitor (Marquette Solar 8000i; GE Medical Systems, Milwaukee, WI, USA) at 400 Hz (NIDAQPad-6015; National Instruments, Austin, TX, USA). For the present analyses, these data were exported as.txt-files to LabChart for further analysis. Cardiac stroke volume was measured by esophageal Doppler monitoring (CardioQ; Deltex Medical, Chichester, UK) and exported for each heartbeat from its serial output.

### PIVA analysis

Short-time Fourier transform (STFT) on peripheral venous pressure waveforms was performed in LabChart using fast Fourier transform (FFT) with a Hann (cosine-bell) data window and 50% overlap. In the LBNP-cohort, FFT windows of 8k datapoints (corresponding to 8.192 s) were used, whereas in the GA-cohort, a 4 K window was used (corresponding to 10.24 s) due to lower sampling frequency, to approximate the window time of the LBNP–cohort.

After performing STFT, the frequencies corresponding to between 0.8 and 1.2 times the heart rate were extracted, and the maximal amplitude within these extracted frequencies was found for each time window. This amplitude was defined as the fundamental frequency of the cardiac component of the peripheral venous pressure waveform; PIVA_F1_.

### Statistics

The changes in PVIA_F1_ and other hemodynamic data in the LBNP-cohort were analyzed in R/Rstudio, using linear mixed models to account for repeated measurements within participants using the “nlme” and “multcomp” packages, with participant as a random effect [17]. For the analysis of association between PIVA_F1_ and LBNP, PIVA_F1_ amplitude was entered as the response variable and LBNP level as a continuous explanatory variable.

For the GA-cohort, the ability of PIVA_F1_ amplitudes to predict fluid responsiveness during head-down tilt was analyzed in a ROC-plot and logistic regression. In a meta-analysis [18], a change in cardiac output of 10 ± 2% with a passive-leg raise was found to best predict fluid responsiveness, and a change in stroke volume of 10% was therefore used as a threshold to define responders in this analysis. However, as this threshold is somewhat arbitrary and forces the dichotomization of the outcome, we also performed an analysis using ordinary least square regression with change in stroke volume as a continuous explanatory variable.

*P* < 0.05 was considered statistically significant in the analyses from both cohorts.

## Results

The characteristics of the two study cohorts are presented in Table 2.

**Table 2:** Characteristics of the study cohorts

	LBNP-cohort (*n* = 15)	GA-cohort (*n* = 20)
Age, years	24 (22–28)	60 (13)
Height, cm	175 (8.7)	173 (9)
Weight, kg	73 (7.1)	75 (16)
Female gender, n (%)	7 (47)	7 (35)
ASA physical status I/II/III, n (%)		1/16/3 (5/80/15)
Tidal volume/weight, mL/kg		7.8 (7.5–8.5)
NA infusion; 0/0.03/0.04/0.05 (µg/kg/min), n (%)		15/2/1/2 (75/10/5/10)
Comorbidities		
Hypertension, n (%)		8 (40)
Type II Diabetes, n (%)		3 (15)
Chronic obstructive lung disease, n (%)		2 (10)
Ischemic heart disease, n (%)		1 (5)

Data are mean (SD) or median (25th- 75th percentiles) unless otherwise stated

ASA, American Society of Anesthesiologists physical status classification; NA, noradrenaline

### PIVA in LBNP-cohort

**Fig. 2:**
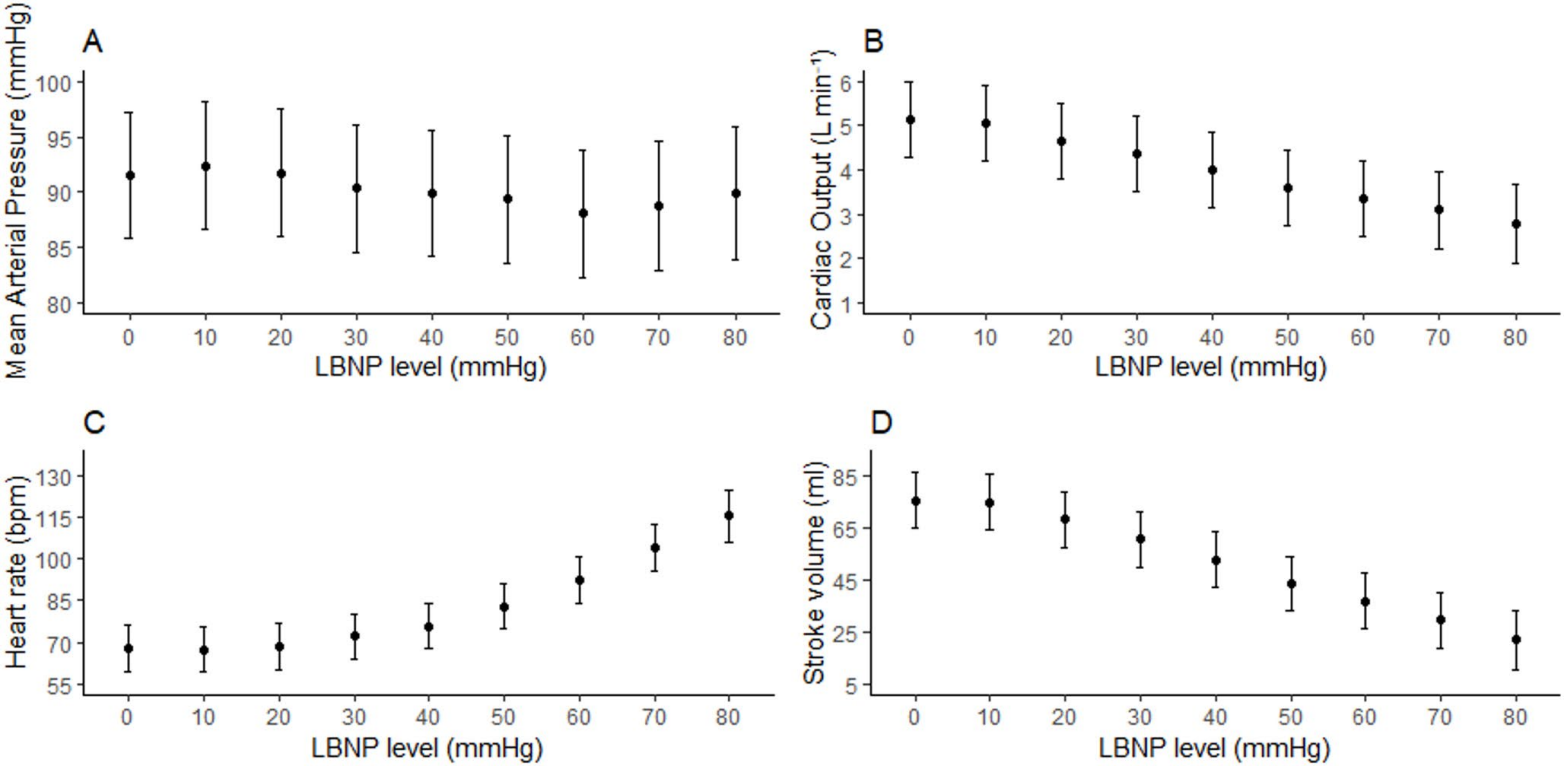
Estimates with confidence intervals for mean arterial pressure (**A**), cardiac output (**B**), heart rate (**C**) and stroke volume (**D**) during lower body negative pressure (LBNP) from 0 to 80 mmHg

**Fig. 3:**
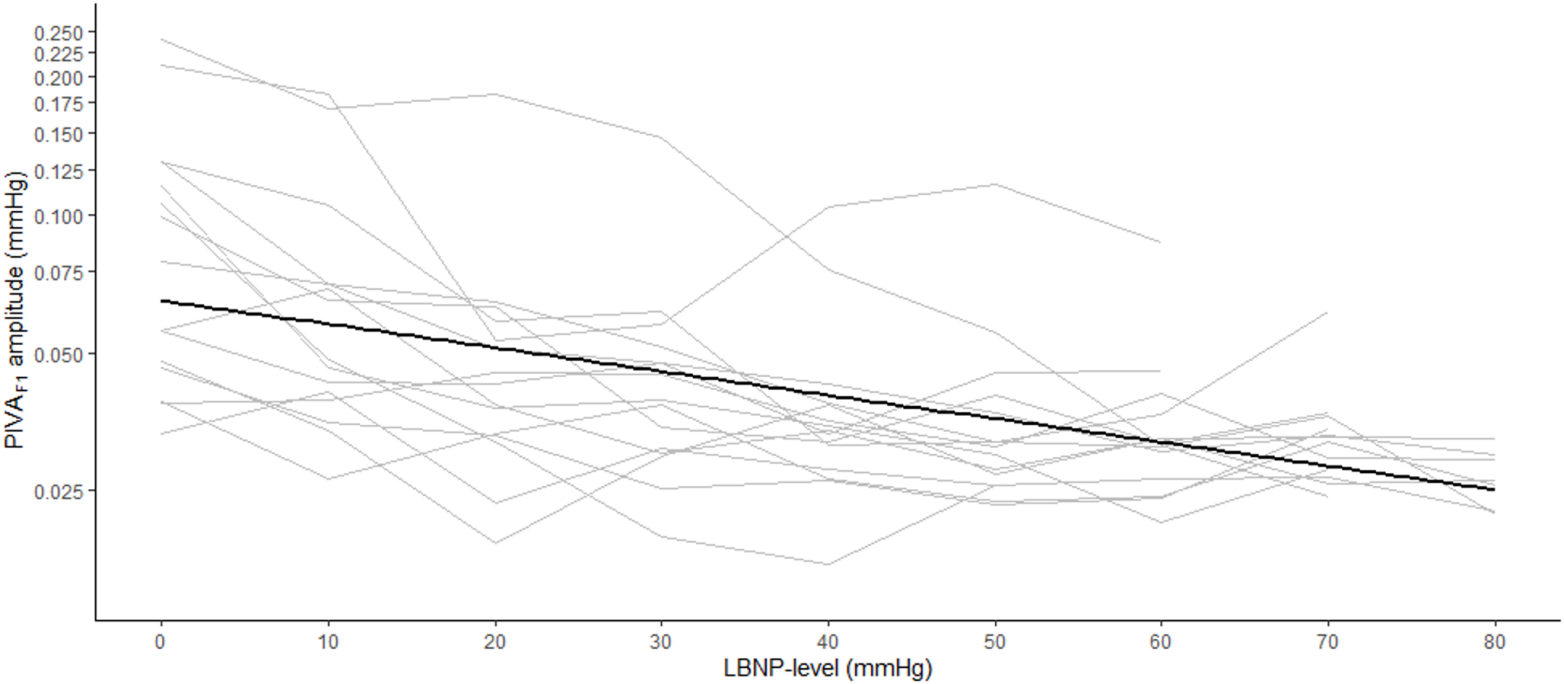
PIVA_F1_ (mmHg) plotted against increasing levels of lower body negative pressure (LBNP) from LBNP 0 to 80. Thin lines represent individual participants. Thick line is regression estimate of the association between PIVA_F1_ and LBNP. Although analyzed after log(e)-transformation, note that the values on the y-axis are back-transformed to original scale for clarity. PIVA_F1_, fundamental frequency of peripheral venous pressure analysis; LBNP, lower body negative pressure

Figure 2 shows the effects of LBNP on the different general hemodynamic measurements. At baseline, PIVA_F1_ was median (1st, 3rd quartile) 0.08 mmHg (0.05 to 0.12). Changes in PIVA_F1_ during progressive LBNP are shown in Fig. 3. Due to heteroscedasticity when analyzed on the original scale, PIVA_F1_ was log_e_ – transformed for analyses.

We found small but statistically significant reductions in log_e_(PIVA_F1_) with increasing LBNP. Log_e_(PIVA_F1_) amplitude at baseline (LBNP 0) was − 2.6 (95% confidence interval (CI): −2.8 to −2.4), decreasing with − 0.11 (95% CI: −0.14 to −0.09, *P* < 0.001) for each 10 mmHg LBNP level.

### PIVA in GA-cohort

**Fig. 4:**
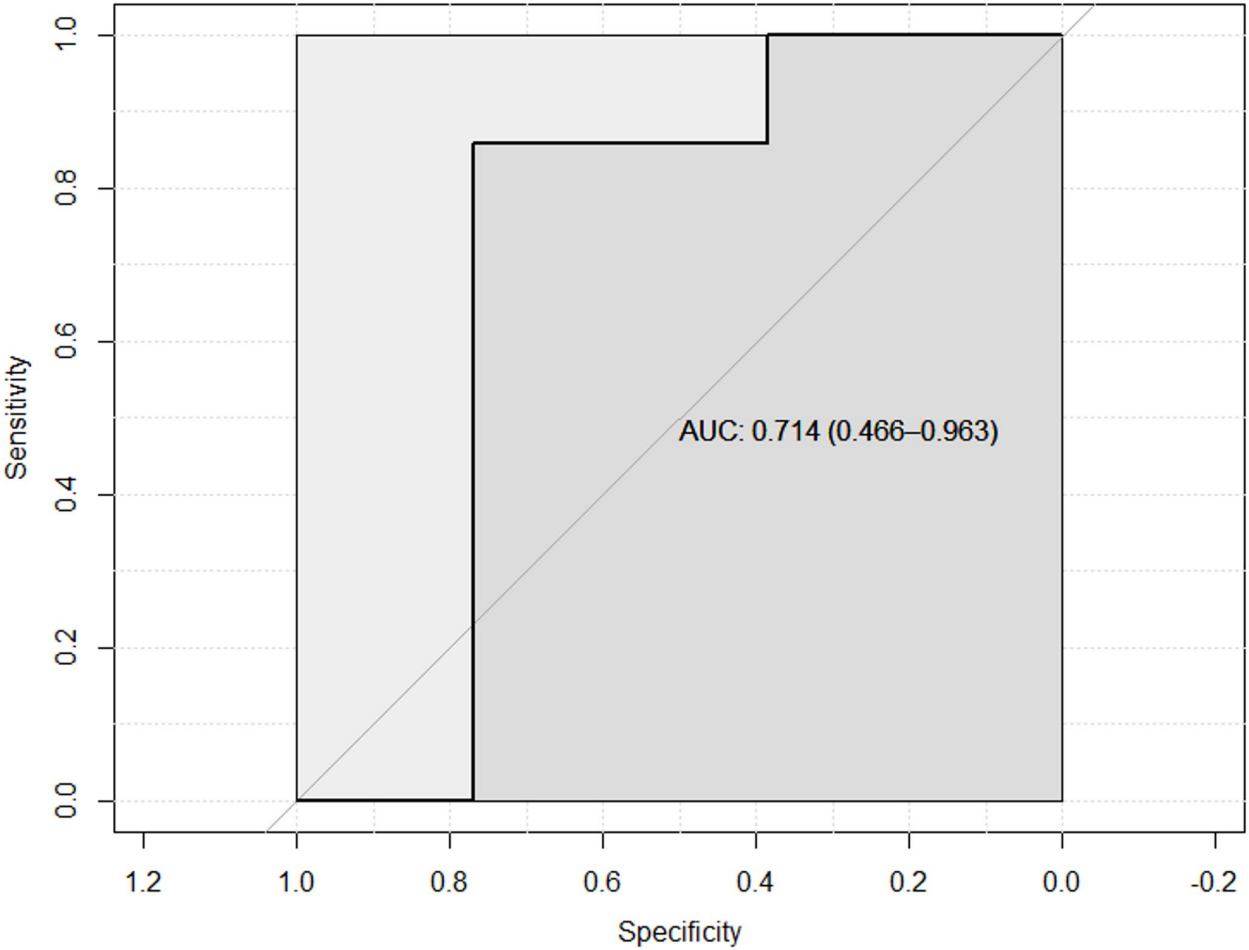
ROC-plot and area under the curve (AUC) with 95% confidence intervals for the ability of PIVA_F1_ during horizontal position to predict a change in stroke volume of 10% with a head-down tilt. PIVA_F1_, fundamental frequency of peripheral venous pressure analysis

**Fig. 5:**
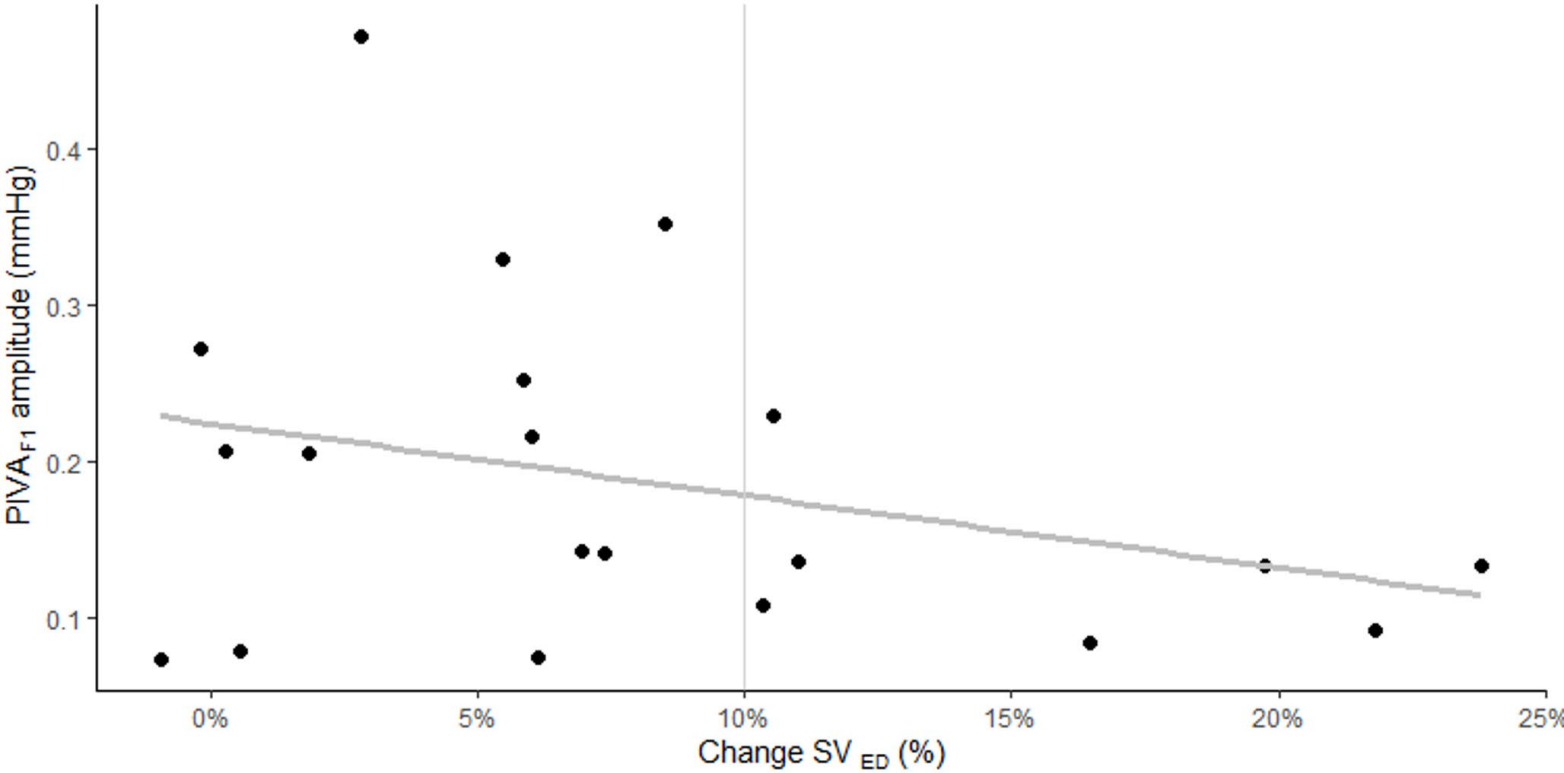
Scatterplot comparing PIVA_F1_ and changes in stroke volume induced by head-down tilt. Thick grey line is regression line. Vertical thin grey line represents separation at 10% change between responders and non-responders to head-down tilt. PIVA_F1_, peripheral intravenous pressure waveform analysis – fundamental frequency amplitude; ED, esophageal doppler

Before head-down tilt, PIVA_F1_ was mean (SD) 0.19 (0.11). In the ROC-plot (Fig. 4), we did not find PIVA_F1_ to be able to predict a change in SV of 10% with the succeeding head-down tilt [AUC 0.71 (95% CI 0.47 to 0.96; *P* = 0.11)]. Correspondingly, when using change in stroke volume as a continuous outcome (Fig. 5) we did not find a statistically significant association between PIVA_F1_ and relative change in stroke volume with head-down tilt (regression slope coefficient − 0.46, 95% CI: −1.15 to 0.23; *P* = 0.18). With the log(e)-transformation of PIVA_F1_, the regression slope coefficient was − 2.1, 95% CI: −5.7 to 1.5; *P* = 0.24. When substituting change in pulse pressure for change in SV, the regression slope coefficient was 0.20, 95% CI: −1.0 to 1.4; *P* = 0.74.

**Fig. 6:**
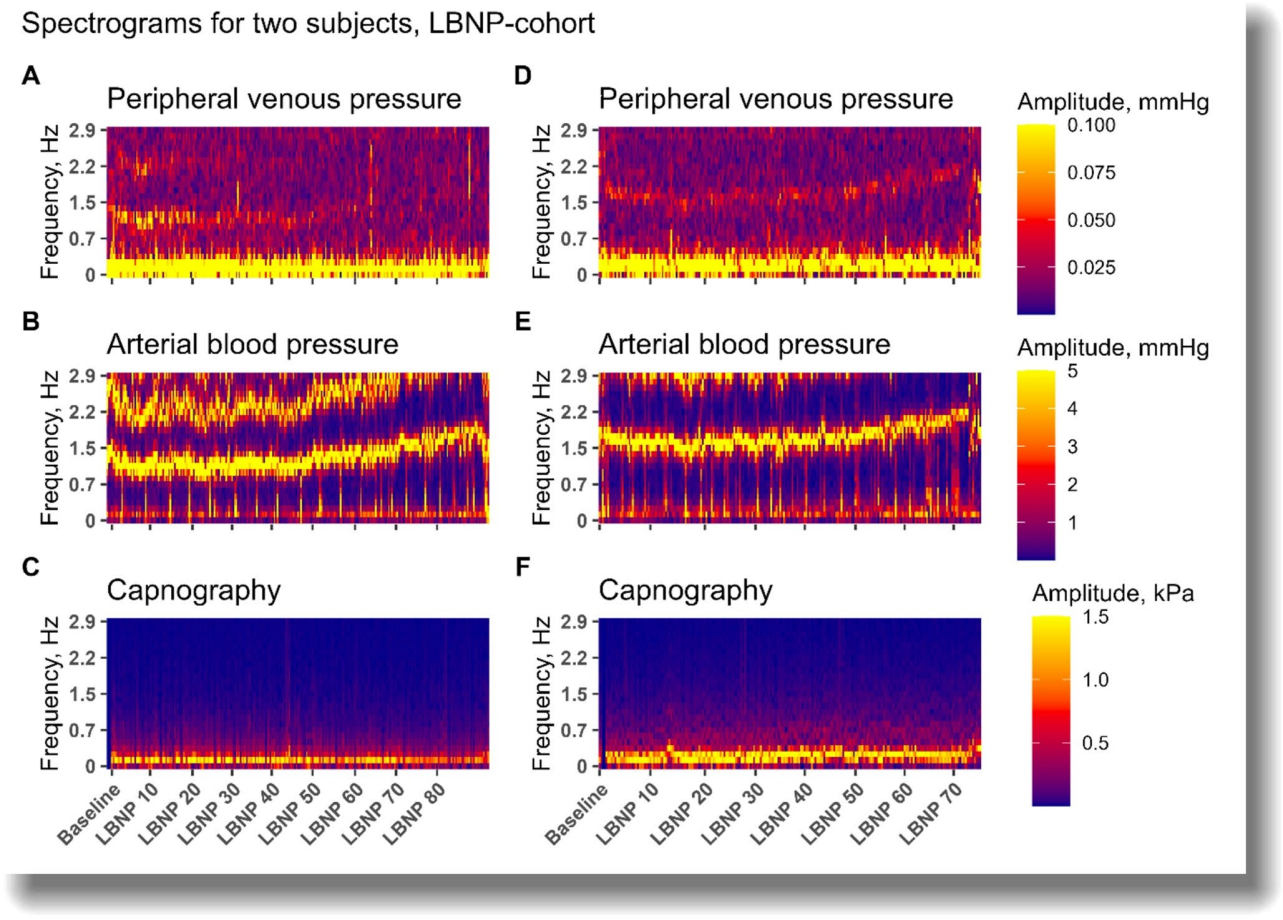
Spectrograms of peripheral venous waveforms (**A**, **D**), arterial pressure waveforms (**B**, **E**) and capnography (**C**, **F**) for two subjects during LBNP. Time on x-axis with LBNP-levels. The subject in the right panels experienced hemodynamic decompensation at LBNP 70, prompting relief of LBNP. LBNP, lower body negative pressure

Figure 6 are spectrograms illustrating the amplitudes over time of frequencies between 0 and 3 Hz from two subjects during LBNP. In both subjects, a clear signal is found as expected in capnography at approximately 0.25 Hz, representing respiration. A clear blood pressure signal is also found in the frequency of the heart rate, and the second harmonic (at twice the frequency) is also partly displayed. For the peripheral venous pressure signal, the amplitude corresponding to respiration is the most prominent in both subjects. At the frequency of the heart rate, a fainter signal is seen at baseline, seeming to fade with increasing LBNP in the subject presented to the left. It should be noted that in many subjects, a clear signal in the peripheral venous spectrogram could not be found.

Median PIVA_F1_ was 0.08 mmHg in the LBNP-cohort at LBNP 0 and 0.14 mmHg in the GA-cohort before head-down tilt (Wilcoxon test *P* = 0.017).

## Discussion

In the present analysis, we found statistically significant reductions in PIVA_F1_ during simulated blood loss, but PIVA_F1_ did not predict increasing stroke volume during head-down tilt in general anesthesia.

Traditionally, clinical hemodynamic monitoring has focused on the heart and arterial part of the systemic circulation, paying less attention to the venous side. However, there is an increasing interest in the venous part of the circulation in critically ill patients [19]. Although the value of monitoring central venous pressure per se for guiding fluid therapy has been heavily criticized [20], use of more advanced analyses of the venous pressures has been advocated [21, 22].

The central venous pressure displays a characteristic waveform pattern, with variations both with each cardiac and respiratory cycle [23]. As nearly all patients are equipped with a peripheral venous cannula during surgery, acute and intensive care, analysis of the peripheral venous pressure waveform has been explored [24, 25]. The peripheral venous pressure waveform may resemble that of the central veins, though its oscillations are generally more dampened [24]. Whether central pressure oscillations are transmitted to the periphery may depend on the presence of venous valves and, importantly, whether there is a continuous fluid column in via noncollapsed veins from the right atrium to the peripheral measurement site. This may be more prevalent during positive pressure ventilation, as right atrial pressure relative to the atmosphere is increased [26]. For the same reason, peripheral veins are presumably also more prone to collapse during hypovolemia.

In the present study we therefore present data during preload reductions in spontaneously breathing subjects as well as during preload increase in patients during positive pressure ventilation. At baseline, median PIVA_F1_ in the LBNP-cohort breathing spontaneously was about half of that of the GA-cohort receiving positive pressure ventilation. This difference may be due to positive pressure ventilation imposing larger venous oscillations as well as higher intrathoracic pressures contributing to a continuous column of blood to the periphery by which pressure oscillations may be transmitted.

The agreement between central and peripheral venous pressure was not found to differ between different peripheral venous cannulation sites in children during positive pressure ventilation [27]. However, in that study, measurements without a presumed continuous fluid column from the right atrium (all peripheral on extremities) were discarded. The choice of peripheral vein used for pressure monitoring may therefore be of importance. The spectrogram presented in the study by Alian et al. [11], resembles that of the subject to the left in Fig. 5, with disappearance of oscillations with increasing LBNP. This disappearance may be compatible with a loss of a continuous fluid column. However, in this study [11] the anatomical location of the peripheral venous cannulation was not specified, and it is possible that a continuous fluid column is more prone to be lost at lower LBNP when placed on e.g. on the dorsum of the hand compared to an antecubital vein, as in the present analysis.

It should be noted that we extracted the maximal amplitude of the frequencies in the vicinity of the heart rate. We did not search for a defined peak or indeed require the presence of one for extracting the maximal amplitude. Therefore, if a defined peak corresponding to oscillations in the heart rate frequency band disappeared during the measurements – or indeed was never present – a value would nevertheless be extracted. Furthermore, the PIVA_F1_ does only capture the fundamental frequency of a cardiac-synchronous oscillation. The values of PIVA_F1_ may therefore be smaller than the amplitude of the original venous pressure signal, as harmonics will also contribute to the total amplitude.

We used two different methods to relate PIVA_F1_ to volume status. In the LBNP-cohort, hypovolemia was induced in the experimental model and PIVA_F1_ was related to the LBNP-level. At baseline, we assumed that the healthy volunteers were normovolemic, with increasing hypovolemia as LBNP was increased. In the GA-cohort, we assumed that the response to a head-down tilt reflected the volume status preceding the tilt. Placing a patient in the Trendelenburg position increases cardiac stroke volume on a group level [28]. Further, by placing patients in the operating room in the Trendelenburg position, the change in left ventricular outflow tract velocity time integral predicts fluid responsiveness [29]. Therefore, the further down on the of the Frank-Starling-curve, the more cardiac stroke volume is expected to increase when placed in the Trendelenburg position.

In the LBNP-cohort, we found that PIVA_F1_ decreased as LBNP increased, corresponding to PIVA_F1_ being able to track compensated hypovolemia on a group level in spontaneously breathing subjects. However, although the association was statistically significant, any clinical significance remains to be elucidated as the changes were small. In the GA-cohort, lower PIVA_F1_-values tended to be associated with larger increases in stroke volume in response to head-down tilt, but this was not statistically significant, and when dichotomizing the change in stroke volume, PIVA_F1_ did not significantly predict volume responsiveness. Hence, we found a statistically significant association between hypovolemia and decreasing PIVA_F1_ in the LBNP-cohort, but not a statistically significant association between low volume status and low PIVA_F1_ in the GA-cohort. The lack of a statistically significant association between PIVA_F1_ and volume status in the GA-cohort could be due to smaller relative variations in volume status in the GA-cohort compared to the LBNP-cohort.

The use of PIVA in clinical settings shows potential but comes with limitations. Whether such small changes can be detected with optimal instrument precision in potentially bleeding patients or in operative settings remains uncertain and calls for further research.

### Methodological considerations

In this study, we evaluated changes in PIVA_F1_ in both spontaneously breathing healthy individuals and mechanically ventilated patients. The limited number of study participants and patients (15 and 20, respectively) should however be considered an important limiting factor. There may be group differences between females and male participants, as well as between the patients in GA-cohort. Five patients received noradrenaline infusion. It is not known whether vasoactive drugs affect PIVA signal.

LBNP is considered a valid model to study many physiologic effects of hypovolemia, and has been extensively used to do so [9]. It does however not cause any tissue damage which limits its validity for trauma patients.

Based on the distribution of the residuals, we log(e) transformed PIVA_F1_ in the LBNP cohort but not in the GA- cohort, complicating the interpretation of the results and comparison with other studies. The requirement may also highlight the complexity of PIVA_F1_ as a potential analysis tool in the future. We attempted to perform the analysis in the GA-cohort with PIVA_F1_ log(e)-transformed, but this also led to a statistically non-significant association with the change in SV and thus did not change the conclusion.

There are inherent limitations to measurements of stroke volume using esophageal Doppler. The velocity-time integrals are measured, but aortic diameter and angle of insonation are based on reference values. However, if the latter two are constant within one patient, they should not affect the relative changes measured, as used in the present analysis. The Doppler-probe was fixed to the endotracheal tube to prevent any change in depth or rotation as the head-down tilts were performed. However, a change in the position of the esophagus relative to the aorta with the position change cannot be excluded. We therefore substituted the change in pulse pressure for the change in SV, thereby using pulse pressure as a proxy for SV. This did, however, not change the conclusion of a statistically non-significant association with PIVA_F1_.

The peripheral venous cannula was used solely for measuring pressure, and no fluid was infused in this or other cannulas during data recording. Further studies are needed to elucidate any effects on PIVA_F1_ of simultaneous fluid infusion in the same or other extremities.

We dichotomized the change in stroke volume with a threshold of 10%. This threshold is based on previous literature [18], but should also reflect the precision of the method. We have previously found a precision of 4.8% giving a least significant change of 6.9% for 30 s measurements with esophageal Doppler [30], and our method should therefore fit well with this threshold.

The apparent discrepancy between the findings in the LBNP and GA-cohorts may have been caused by a spectrum effect [31]. The variability in volume status was presumably small in the GA-cohort compared to the LBNP-cohort, which may have affected the AUC-value of the ROC-plot [32].

## Conclusion

PIVA_F1_ offers a potential minimally invasive method for volume assessment. In the current study, we found a statistically significant reduction in PIVA_F1_ during experimental hypovolemia. However, PIVA_F1_ was not associated with changes in stroke volume in response to head-down tilt in patients under general anesthesia. Further studies are required to validate PIVA_F1_ for assessing volume status and guiding fluid therapy in clinical settings.

## Data Availability

Data will be available from the corresponding author upon reasonable request and agreement from the Data Protection Officer.
